# Microscopic Varicocelectomy Significantly Decreases the Sperm DNA Fragmentation Index in Patients with Infertility

**DOI:** 10.1155/2014/695713

**Published:** 2014-03-10

**Authors:** Teoman Cem Kadioglu, Emin Aliyev, Murad Celtik

**Affiliations:** Department of Urology, Istanbul School of Medicine, University of Istanbul, 34365 Istanbul, Turkey

## Abstract

*Background*. Varicocele is associated with high levels of DNA damage in spermatozoa due to oxidative stress and elevated levels of sperm DNA fragmentation, which has been currently proposed to be an essential additional diagnostic test to be recommended for patients with clinical varicocele. The aim of this study was to evaluate the parameters of semen and the DNA fragmentation index (DFI) in patients with varicocele before and after varicocelectomy. *Methods*. The details of 92 consecutive patients were retrospectively analyzed from January 2010 to December 2012. The sperm samples were evaluated according to the World Health Organization Guidelines. Sperm DNA damage, characterized as DFI, was evaluated by sperm chromatin structure assay using flow cytometry. *Results*. There was a statistically significant improvement in the semen concentration, the total motile count, the total normal sperm count, and the sperm DNA fragmentation index (DFI; the percentage of sperm with denatured DNA) after varicocelectomy. There was a large decrease in DFI from a preoperative mean of 42.6% to a postoperative mean of 20.5% (*P* < 0.001). A higher preoperative DFI was associated with a larger decrease in postoperative DFI, and significant negative correlations were observed between the DFI and sperm motility (*r* = −0.42, *P* < 0.01). *Conclusion*. Our data suggest that varicocelectomy can improve multiple semen parameters and sperm DNA damage in infertile men with varicocele. The patients with preoperative defects in those parameters showed greater improvement postoperatively. Further research in this area is needed to understand the exact mechanisms of DNA damage in infertile men with varicocele.

## 1. Introduction

Varicocele is the dilatation of the pampiniform plexus caused by the reversal of venous blood within the spermatic veins [1]. Varicocele is an underlying problem in male infertility. The prevalence of varicocele has been reported to be as high as 10~15% in the general population, 30~35% in men with primary infertility, and 69~81% in men with secondary infertility [1]. Many studies have been conducted to explain the pathophysiology of testicular dysfunction occurring with varicocele. The exact mechanism of infertility caused by varicocele is not understood completely. The most likely explanation is that germinal cell dysfunction is secondary to hypoxia from the obstruction of small vessels and venous stasis [2]. The back flow of adrenal and renal metabolic products through the left internal spermatic vein, an increase in scrotal temperature, and endocrinological changes are other explanations that have been proposed to explain infertility from varicocele [3–5].

Additional hypotheses on the mechanisms of infertility in men with varicoceles are associated with increased oxidative stress and decreased antioxidant capacity. This parameter has been linked to sperm DNA damage, such as DNA fragmentation, and correlated with the reduced ability of spermatozoa to fertilize oocytes in assisted reproduction techniques and normal fertility [6, 7].

The sperm DNA fragmentation index (DFI: percentage of sperm with denatured nuclei) is a potential parameter for fertility investigation [8, 9]. DNA fragmentation, which is generally due to increased oxidative stress and decreased antioxidant capacity, is the separation or breaking of DNA strands into pieces. Spontaneous or accidental DNA fragmentation is fragmentation that gradually accumulates in a cell. The degree of DNA fragmentation can predict outcomes for in vitro fertilization (IVF) and by extension intracytoplasmic sperm injection (ICSI) [10, 11]. Main unit of measurement for DNA fragmentation is “DFI” [11]. A DFI of 20% or more significantly reduces the success rates after ICSI [11].

Recent studies indicate that surgical repair of varicocele significantly improves sperm DNA quality [12]. Therefore, the aim of the study was to compare the levels of common semen parameters, as well as the extent of DNA damage, assessed as DFI measured by flow cytometry, before and after subinguinal microsurgical varicocelectomy in infertile men with varicocele.

## 2. Materials and Methods

### 2.1. Patient Selection

A retrospective analysis was performed on 92 consecutive infertile men who underwent subinguinal microsurgical varicocelectomy at our andrology institution from January 2010 through December 2012. Men presenting at our clinic with 1 year or more of infertility, a clinically palpable varicocele (classified as Grades 1, 2, and 3 varicocele), and abnormal semen parameters (2 or more semen samples) were received as candidates for varicocele repair. Men with systemic or endocrine disease, cryptorchidism, hypogonadism, genital infection, cigarette smoking habit, alcohol or drug abuse, hormonal treatment and azoospermic patients were excluded. All the types of microsurgical varicocelectomy were performed by the same surgeon (T.C.K.), as previously described in detail elsewhere [13]. The protocol was approved by the internal institutional review board, and informed written assent was obtained from each participant.

### 2.2. Sperm Collection and Semen Analysis

Each patient was instructed to abstain from sexual activity for 72 h and self-collect a semen sample, which was analyzed within 1 h of collection. Manual semen analysis was performed for sperm concentration, total sperm count, and percent motility and viability by 4 different dedicated technicians using the World Health Organization (WHO) normal values based on the WHO 2010 reference limits [14].

### 2.3. Measurement of Sperm DNA Damage

Sperm DNA fragmentation was quantified using the terminal deoxynucleotidyl transferase-mediated fluorescein-dUTP nick end labeling assay kit (Apo-Direct; Pharmingen, San Diego, CA). After fixation with 3.7% paraformaldehyde for 30 minutes on ice, the spermatozoa were washed and resuspended in ice cold 70% ethanol, followed by another resuspension in phosphate-buffered saline. The specimens were centrifuged at 1600 rpm for 7 minutes, and the pellet was resuspended for 60 minutes at 37°C in 50 *μ*L of solution containing terminal deoxynucleotidyl transferase enzyme, terminal deoxynucleotidyl transferase reaction buffer, fluorescein isothiocyanate tagged 20-deoxyuridine, 50-triphosphate nucleotides, and distilled water. After centrifugation, the cells were washed twice in a rinse buffer, resuspended in 0.5 mL of propidium iodide/RNase solution, and incubated for 30 minutes in the dark at room temperature in anticipation of the flow cytometry.

### 2.4. Flow Cytometry Analysis

All the fluorescence signals of the labeled spermatozoa were analyzed by a FACScan flow cytometer (Becton Dickinson, San Jose, CA). A total of 10,000 spermatozoa were examined for each assay at a flow rate of <100 cells/second. The excitation wavelength was 488 nm supplied by an argon laser at 15 mW. Green fluorescence (480–530 nm) was measured in the FL-1 channel and red fluorescence (580–630 nm) in the FL-2 channel. Gating was performed to exclude debris and aggregates using a using a 900 nm wavelength and forward-angle light scatter. The DFI was calculated from the ratio of red to total fluorescence on a 1023-channel scale using flow cytometer software version 6.2.4 FlowJo (FlowJo, LLC, Ashland, OR).

### 2.5. Statistical Analysis

The results are expressed as the means ± SD. The differences between the pre- or postvaricocelectomy parameters were estimated using a Wilcoxon signed-rank test. All *P* values were 2-tailed and a *P* value of <0.05 was considered statistically significant. Statistical analysis was conducted using STATA, version 10.1, (STATA Corporation, College Station, TX).

## 3. Results

A varicocele was detected by physical examination and confirmed by Doppler ultrasound in the 92 patients who entered the study. The demographic and clinic findings are provided in Table 1. The mean age of the 92 men was 33.8 ± 3.8 (range: 22–39) years with a mean duration of infertility of 21.6 ± 9.2 months. Eighty patients (84.2%) presented with a varicocele isolated on the left side, and 12 patients (15.8%) had bilateral varicocele. The preoperative follicle-stimulating hormone was measured in 58 patients, and the median value was 4.8 mU/Ml (1.9–25.1). The preoperative testosterone was measured in 29 patients, and the median value was 3.2 ng/dL (1.8–7.3).

The sperm parameters, including volume, sperm count, and motility, are listed in Table 2. Six months after subinguinal microsurgical varicocelectomy, the patients showed higher sperm count, progressive motility, and normal forms compared with baseline (*P* < 0.05). There was a large decrease in DFI from a preoperative mean of 42.6% to a postoperative mean of 20.5% (*P* < 0.001).

Grade 3 varicocele patients were associated with a greater mean improvement in semen count than grades 1 and 2. Higher preoperative DFI was associated with a larger decrease in postoperative DFI and significant negative correlations between DFI and sperm motility (*r* = −0.42, *P* < 0.01).

## 4. Discussion

This study examined the DFI and sperm parameters in patients with different grade varicocele who did not have any identifiable systemic or andrological cause of sperm or DNA abnormalities before and 6 months after surgery. Our result confirmed that patients with varicocele had lower sperm density, total sperm count, motile spermatozoa, and normal form. These patients had increased percentages of spermatozoa with fragmented DNA. After microscopic varicocelectomy, all the semen parameters and DFI significantly improved compared with preoperative values. These results support that microsurgical repair must be considered as a treatment option in infertile men with palpable varicocele, since improvement in DFI is clearly shown to be associated with higher pregnancy rates (spontaneous or with assisted reproductive technique) [11, 15]. However there is some research suggesting that sperm DNA damage was not related to outcomes of IVF or ICSI with own or donated oocytes. Clinical pregnancy and implantation rates seem to be independent of sperm DNA fragmentation [16].

The association between a clinical varicocele and impaired spermatogenesis is well described [1–3]. Varicoceles have been associated with high levels of sperm DNA damage [10–12]. Saleh et al. implicated elevated temperature and the elaboration of reactive oxygen species as potential mechanisms responsible for varicocele-mediated sperm dysfunction and DNA damage [17]. The diagnosis and followup of infertile men are generally performed using semen analysis. Although semen analysis provides valuable information, it has limitations. Semen parameters show biological intra- and interobserver variations [14]. Sperm quality is predominantly evaluated based on motility and morphology. It has been demonstrated that, in infertile men, sperm with normal morphology have high rates of DNA fragmentation [8, 9]. DFI provides additional information about the potential for fertility and shows less biological variation when compared to conventional semen analysis [9].

Several researchers have examined the effect of microsurgical varicocelectomy on sperm DNA damage in infertile men [15, 18, 19]. In a recently published meta-analysis, the overall estimate showed that patients with varicoceles had significantly higher sperm DNA damage than controls, with a mean difference of 9.84% (95% CI, 9.19 to 10.49; *P* < 0.00001) [19]. The meta-analysis also showed that varicocelectomy could improve sperm DNA integrity, with a mean difference of −3.37% (95% CI, −4.09 to −2.65; *P* < 0.00001). Based on the results of the meta-analysis, it was concluded that there was increased sperm DNA damage in patients with varicoceles and that varicocelectomy could be a possible treatment; however, more studies with appropriate controls were needed to confirm these findings [19].

Baker et al. examined 83 subjects who underwent varicocele ligation for a fertility concern [20]. The aim of their study was to identify the preoperative parameters that predict improvement in postoperative semen values and variables that predict pregnancy after surgery. Their study revealed a statistically significant decrease in DFI after varicocelectomy and that a higher preoperative DFI was associated with a greater postoperative decrease. Interestingly, a higher mean postoperative DFI was reported in couples reporting a spontaneous pregnancy (34%) compared to couples reporting conception through IVF/ICSI (17.5%), and this difference reached statistical significance. They did not find a difference in the mean postoperative DFI in couples who were able to achieve a pregnancy compared to couples that reported no pregnancies [20]. The association of DFI and pregnancy was explored by Smit et al. in 49 men with infertility [15]. The authors confirmed an improvement in the mean sperm count, concentration, progressive motility, and DFI after varicocelectomy. The authors noted that an improvement in progressive motility was inversely associated with a postoperative decrease in DFI. After varicocelectomy, 37% of the couples conceived spontaneously, and 24% achieved pregnancy with assisted reproductive techniques. The mean postoperative DNA fragmentation index was significantly higher in the couples that did not conceive spontaneously or with assisted reproductive techniques [15].

In the present study, the patients showed higher sperm count, progressive motility, and normal forms 6 months after subinguinal microsurgical varicocelectomy (*P* < 0.05). Furthermore, there was a large decrease in DFI from a preoperative mean of 42.6% to a postoperative mean of 20.5% (*P* < 0.001), supporting the previously published literature discussed above. However, there were several limitations in this current study. First of all, the retrospective, nonrandomized design of the study and the sample size diminish the impact of the findings. It would be interesting to randomize infertile men with varicocele into surgical treatment versus no treatment groups and assess the DFI as well as other reproductive parameters, especially pregnancy rates, during followup. However this randomization seems to be hard to receive ethical approval at this moment.

As a conclusion, varicocelectomy resulted in a statistically significant improvement in sperm concentration, motility, and DFI. Patients with preoperative defects in those parameters showed greater improvement postoperatively. Sperm DNA damage may affect the quality of ejaculated spermatozoa and may have implications in the fertility of patients. Ongoing studies will hopefully determine whether improved DFI may enable these infertile men to father child spontaneously or by assisted reproduction techniques. Further research in this area is needed to understand the exact mechanisms of DNA damage for infertile men with varicocele.

## Figures and Tables

**Table 1:** Demographic and clinical findings of the patients.

Age	33.8 ± 3.8
BMI	26.2 ± 2.9
Type of infertility *n* (%)	
Primary	71, (74.7%)
Secondary	24, (25.3%)
Distribution of varicocele *n* (%)	
Unilateral	80, (84.2%)
Bilateral	15, (15.8%)
Varicocele grade *n* (%)	
Grade 1	8, (8.4%)
Grade 2	34, (35.8%)
Grade 3	53, (55.8%)
Duration of infertility (months)	21.6 ± 9.2

**Table 2:** Preoperative and postoperative sperm parameters and DNA fragmentation index (DFI).

Variable	Preoperative	Postoperative	P*
Volume (mL)	2.7 ± 1.3	2.9 ± 1.5	N.S.
Count (mil/mL)	34.6 ± 23.8	41.4 ± 15.9	<0.05
Progressive motility (*a* + *b*) (%)	19.9 ± 7.1	39.3 ± 6.4	<0.05
Sperm DFI	42.6%	20.5%	<0.001

*Wilcoxon signed-rank test.
